# A translational model for early childhood intervention: developing, implementing, and scaling-up effective practices

**DOI:** 10.3389/fpubh.2023.1198206

**Published:** 2023-07-07

**Authors:** Kallen R. Shaw, Ramzi G. Salloum, Patricia A. Snyder

**Affiliations:** ^1^School of Special Education, School Psychology, and Early Childhood Studies, College of Education, Anita Zucker Center for Excellence in Early Childhood Studies, University of Florida, Gainesville, FL, United States; ^2^Department of Health Outcomes and Biomedical Informatics, College of Medicine, University of Florida, Gainesville, FL, United States

**Keywords:** translational research, implementation science, early intervention, evidence-based practices, early childhood

## Abstract

Early intervention (EI) researchers (i.e., those focused on children birth to age 3 and their families who experience early vulnerabilities) often engage in translational research and implementation science at the intersection of public health, pediatrics, and EI. There is currently a significant research-to-practice gap in EI despite ongoing efforts to close it. Translational research and implementation science are promising approaches to promote transdisciplinary collaborations among researchers and to move EI research into practice, thus supporting positive outcomes for young children and families. This commentary proposes a contemporary alignment of translational research phases for EI. Two literature reviews served to inform development of this alignment: (1) a narrative literature review identified existing applications of translational phases to EI; and (2) a rapid review identified examples of existing behavior-focused translational models across disciplines. Several case examples of current translational research being conducted in EI are discussed and classified according to their respective translational phase. The proposed alignment and case examples provide a basis for transdisciplinary conversations among those working across the various fields and disciplines relevant to EI research. A shift in EI research to reflect a translational and implementation focus will help bridge the research-to-practice gap and, most importantly, speed the movement of scientific evidence into real-world contexts to positively impact young children and families.

## 1. Introduction

Researchers in early childhood often focus on intersections between efficacy and effectiveness research as they study programs and practices. In doing so, these researchers support the fidelity of practitioner implementation of evidence-based practices (EBPs) in authentic (i.e., real-world) contexts, and promote positive outcomes for young children and their families. Within the broader context of early childhood research, some researchers focus on young children birth to age three and their families who experience early vulnerabilities—an area which is often referred to as early intervention (EI) research. Despite ongoing efforts to close it, a significant research-to-practice gap persists in EI (1, 2). This gap is not unique to the field of EI but is ubiquitous across fields that intersect with EI, including public health and pediatrics (3). Advances in translational research and implementation science hold great promise for bridging this research-to-practice gap. The purpose of this commentary is to propose a contemporary translational research framework that might be useful for researchers, practitioners, and other professionals in EI for advancing transdisciplinary dialogue among those working at the intersections of public health, pediatrics, and EI to support young children and families.

Translational research phases typically describe a bench-to-bedside path which moves from basic or preclinical research, through clinical trials, and ends with a focus on public health and population-level outcomes (4). For EI, an application of this process in the present commentary will focus on the development and movement of EI research from initial stages (e.g., examining key characteristics of effective practice) through sustainability or scale-up (e.g., dissemination and implementation within state- or national-level programs). Translational research has primarily been applied to issues of public health and medicine but has not yet been widely applied in EI. Cancer research is often identified as an example of moving new drug or treatment discoveries through the translational spectrum to implementation at bedside and beyond (5, 6). Another modern example is the rapid development of vaccines and treatments in the COVID pandemic, which included a focus on delivering interventions and care in a timely manner for clinicians and patients (7–9).

While translational research is key for explaining how sources of evidence move from identification into practice (10), it is equally important to consider how implementation processes occur in real-world contexts. The discipline of implementation science is focused on identifying, assessing, and scaling-up evidence-based practices (EBPs) (11, 12). For EI, implementation science frameworks, principles, and strategies have been explicitly designed to support authentic intervention agents (i.e., individuals a child and family would typically interact with) to effectively deliver evidence-based strategies in the natural settings in which young children and families interact and spend time (13). The services and supports offered by EI include practices used directly with the child by providers (e.g., speech therapists, physical therapists) and, more recently caregiver-mediated interventions intended to enhance provider and caregiver capacity to support children’s development and learning in daily routines and activities.

Of particular relevance for translational research in EI is the consideration of pragmatic and hybrid (i.e., effectiveness-implementation) trials. Pragmatic trials aim to examine intervention implementation and effects under real-world conditions and serve as a critical part of the pathway bridging research to practice. Recent examples of EI pragmatic trials include: (a) a randomized clinical trial (14) implemented in routine health settings (i.e., well-child visits and nutritionist visits) which examined the effects of a health information technology intervention on improving care coordination between primary care physicians and Women, Infants, and Children (WIC) nutritionists; and (b) a multisite, randomized controlled trial (15) implemented in routine health settings (i.e., National Health Service) which examined the effectiveness of a video-feedback intervention to promote positive parenting and sensitive discipline. Considerations of the link between effectiveness and implementation may occur through hybrid trials as suggested by Curran et al. (16). These hybrid trials intend to simultaneously gain rapid understandings of clinical and implementation outcomes, thus informing activities in the T4 phase. Recent examples of EI hybrid trials include: (a) a hybrid type I trial (17) (i.e., emphasizing the effectiveness outcome) conducted in a routine health setting (i.e., pediatric practice) which examined intervention effects and collected implementation data of an early literacy promotion program initiated during the newborn period; and (b) a protocol for a hybrid type II trial (18) (i.e., equal emphasis on effectiveness and implementation) which evaluates a lifestyle intervention aimed to address risk factors for overweight and obesity among expecting mothers and their children. For this commentary, we are delimiting our framework alignments across funding agencies up through T3 pragmatic and hybrid trials, recognizing there is a need to further align up through T4 for behavioral interventions.

As EI research continues to mirror and extend the translational work being done in public health and pediatrics, those working to support young children and families will benefit from a shared understanding of how translational research principles are applied in EI. Thus, the objectives of this commentary are to: (a) describe how the phases of translational research apply to EI; (b) offer a proposed model for positioning EI research on the translational spectrum; and (c) review three case examples of recent translational research being conducted in EI.

## 2. Literature review

A narrative literature review was conducted to identify definitions of translational phases through the lens of EI research. Resources considered for review included articles, book chapters, and gray literature, and were included if they: (a) described an application of the translational research phases as proposed by the University of Arkansas for Medical Sciences (UAMS) (4) and comprehensively defined by Surkis et al. (19) to an area of EI; or (b) offered definitions of studies and trials which are used to build evidence for and evaluate interventions throughout translational phases. Resources were included only if they specified connections to seminal translational research articles [e.g., Woolf (20)] and offered definitions or examples for each translational phase.

A secondary, rapid review was conducted to identify existing behavior-focused translational models or frameworks from broader health-related fields. Reviews were conducted using electronic databases (i.e., PubMed, Embase, PsycINFO) and reviews of print sources. It is important to note that these reviews were not exhaustive; rather, the aim was to identify prime examples of behavior-focused translational models across health-related fields (e.g., public health) to inform the translational application to EI. Models which offered proposals for moving from development to implementation of EBPs were included.

### 2.1. Narrative review

The narrative review identified one book chapter (21) which aligned translational research phases with EI. A report from a United Kingdom-based organization was also identified but excluded from this review because the research focused on identifying constructs that are critical for translation of education research, rather than offering definitions for or alignments of translational phases (22). Additionally, given the crossover of funding sources in EI due to the transdisciplinary nature of the work, requests for application (RFAs) and project structures from the Institute of Education Sciences (IES) were considered in the development of this commentary (23–25). These RFAs are key for aligning IES trials in the education sciences with the National Institutes of Health (NIH) clinical trials (26) in the US as part of understanding the translational process for EI research currently funded by IES. Lastly, the trial and funding structure from the Medical Research Council (MRC) (27), a United Kingdom stronghold for translational and clinical research, was included to expand these translational alignments beyond NIH.

The Trivette and Dunst book chapter (21) was the only identified source that provided an alignment with the translational research phases as proposed by UAMS (4) and defined by Surkis et al. (19). Four translational phases were described for EI: Type 1 involves the development of EBPs and research-informed intervention procedures; Type 2 involves the use of evidence-based implementation and intervention practices by professionals and families; Type 3 involves evaluating the impacts of EBPs when used routinely in everyday intervention settings; and Type 4 involves dissemination, diffusion, and scale-up of effective EBPs. Trivette and Dunst referenced the Woolf (20) definition of translational research processes and used this definition to guide the translational framework they proposed for EI.

The IES RFAs and project structures (23–25) offered definitions for four different types of trials. Exploration trials serve to identify relationships across child-, professional-, school-, and policy-levels with education outcomes in addition to identifying factors which influence these relationships. Development and innovation trials involve the formation of promising interventions and demonstration of initial outcomes from such interventions. Initial efficacy and follow-up trials are implemented under controlled conditions and intend to study intervention efficacy and test the longer impacts of interventions which have been shown to have positive impacts in previous trials. Effectiveness trials involve implementation of an intervention under routine conditions by authentic intervention agents; these studies occur in authentic contexts. The definitions provided in these RFAs align with NIH and MRC organization of clinical trials and, together with the Trivette and Dunst (21) chapter, informed the alignment of translational phases for EI described in the present commentary.

### 2.2. Rapid review

The rapid review identified two frameworks that offered a behavior-focused translational model. First, the Obesity-Related Behavioral Intervention Trials (ORBIT) model was developed to facilitate the development of behavioral interventions to prevent or manage chronic diseases (28). Model development was informed by existing guidelines for drug development and previous efforts related to translation of behavioral interventions. The ORBIT model provides guidance on the pathway of translating basic behavioral science by clarifying the chain for collecting evidence to ensure an intervention is ready for testing in a Phase III efficacy trial. The model also encourages a transdisciplinary approach and flexibility in methodologies for intervention development. Second, the Science of Behavior Change (SOBC) framework aims to identify and understand the mechanisms behind behavior change (29). The SOBC framework strives to produce better evidence for behavior change science through three major steps: (a) identifying the mechanism; (b) measuring the mechanism; and (c) influencing the mechanism. Identification and understanding of these core mechanisms will improve researchers’ knowledge of how to support effective behavior change across applications of translational research. Use of the SOBC framework will also support more coordinated efforts across fields to understand and evaluate behavior change mechanisms, in conjunction with implementation support strategies rooted in implementation science.

## 3. Findings

Together, the identified literature provided a baseline to inform a contemporary alignment of the translational phases of EI research and situate it within a translational pathway. From the narrative review of the literature, including the initial work of Trivette and Dunst (21) and the trial types defined by IES (23–25), a basis was gathered for identifying parallels between translational research across EI and the typical health-based application settings. From the rapid review, two exemplar models were identified, which offered frameworks for developing and translating behavior-based interventions. Both the ORBIT (28) and SOBC (29) models emphasize the importance of gathering evidence for the behavior mechanisms being studied so they can be appropriately measured, evaluated, and optimized in later phase trials.

In the application of translational phases to the bench-to-bedside pathway, the intervention “active ingredients” are usually drugs, devices, and biologics. NIH clinical trial phases also reflect this focus. As an alternative to this typical application, Table 1 offers an alignment across the translational phases described by Surkis et al. (19) and Trivette and Dunst (21) and an alignment of NIH clinical trials (26), IES trials (23–25), and MRC trials (27) organized by the translational phases. Together, these alignments serve as a basis for describing the movement of EBPs in the field of EI from discovery to implementation by authentic intervention agents in authentic practice settings. It is important to note that the T0–T2 phases focus on efficacy (i.e., studying the intervention under controlled conditions), and the T3–T4 phases focus on effectiveness (i.e., the intervention under real-world conditions).

**Table 1:** Aligning phases of translational research in early intervention across translational phase definitions and funders.

Translational phase	Translational phase definitions		Funding agencies		
	Surkis et al. (19)	Trivette and Dunst (21)	National Institutes of Health (26)	Institute of Education Sciences (23–25)	Medical Research Council (27)
T0: Basic research	**Basic biomedical research** • Identify opportunities and approaches to health issues. • Understand biological, social, and behavioral mechanisms which underlie health or disease. • Includes non-interventional, correlational epidemiologic studies of large datasets.	Not explicated, but the importance of identifying sources of evidence for best practices is noted.	–	**Exploration trials** • Identify relationships between learner-, educator-, school-, and policy-level characteristics with education outcomes. • Understand factors that influence these relationships.	**Experimental medicine panel** Address questions focused on understanding mechanisms. Intend to produce novel understandings of mechanisms and their targets.
T1: Translation to humans	**Translation to humans** • Seeks to move fundamental discovery into health application; provide clinical insights. • New methods of diagnosis, treatment, and prevention. • Trials occur in highly controlled settings.	**Type 1** • Identify characteristics of evidence-based practices using research findings. • Understand how these characteristics can be used to develop evidence-based interventions.	**Phase I trials** *•* Researchers test a treatment in a small group of people for the first time.• Purpose is to learn about safety and identify side effects.	**Development and innovation trials** *•* Development and/or pilot testing of a novel education intervention which intends to produce beneficial impacts on learner outcomes. • Results in a fully developed intervention.	Developmental Pathway Funding Scheme (DPFS)* Early-stage studies in humans. Understand safety and efficacy.
T2: Translation to patients	**Translation to patients** • Highly controlled clinical research studies to analyze optimal effects of intervention, which may lead to the basis for novel clinical application and evidence-based guidelines. • Yields knowledge about safety and efficacy of interventions.	**Type 2** • Use of evidence-based implementation and intervention practices for practitioners and caregivers to produce desired benefits and outcomes. • Promote adoption of evidence-based practices by practitioners and caregivers.	**Phase II trials** • Treatment is given to a larger group of people. • Determine effectiveness and further study safety.**Phase III trials** • Treatment is given to large groups of people to confirm effectiveness and safety.• Compare with standard or similar treatments.	**Initial efficacy and follow-up trials** • Initial efficacy studies of education interventions predicted to have meaningful impacts on education outcomes. • Test longer-impacts of interventions shown to have beneficial impacts in previous studies.	Efficacy and Mechanism Evaluation (EME)* Studies in humans of interventions with established dose/intensity and existing evidence of treatment effect in humans. May result in evidence of efficacy or effectiveness, effect size, or test mechanism of action.
T3: Translation to practice	**Translation to practice** • Development and implementation of evidence-based guidelines, policies, and best practices. • Includes comparative effectiveness trials, pragmatic trials, community-based participatory research (CBPR), dissemination and implementation, clinical outcomes research, post-marketing analysis.	**Type 3** • Evaluate whether the use of evidence-based practices in everyday intervention settings and by typical intervention agents has the same effects as found in primary studies. • Considerations of different contexts and end-users.	**Phase IV trials** *•* After FDA approval of treatment. • Track safety in general population. • Seek more information about benefits. • Understand optimal treatment use.	**Effectiveness trials** *•* Implementation of an intervention under routine conditions. • Level of implementation support will be no greater than what would be typically received if not taking part in the study. • Sample heterogeneity aligns with that of the target population.	**Health Technology Assessment (HTA)** Conduct comparisons of novel interventions with standard of care interventions. Supports interventions which have some evidence of effectiveness.
T4: Translation to communities	**Translation to communities** • Health practice to population health impact; providing communities with optimal interventions. • Scaling-up improved practices and interventions. • Impacts of policy and environmental change. • “Real-world” health outcomes of population health practices.	**Type 4** • Disseminate, diffuse, and scale-up use of evidence-based intervention practices based on information from T1-3. • Promote broad-based understanding of characteristics of EBPs.	–	–	–

*May crossover between T2 and T3 phases depending on the evidence-base collected for a given intervention and the scale of evidence required to establish safety and efficacy.

**Figure 1:**
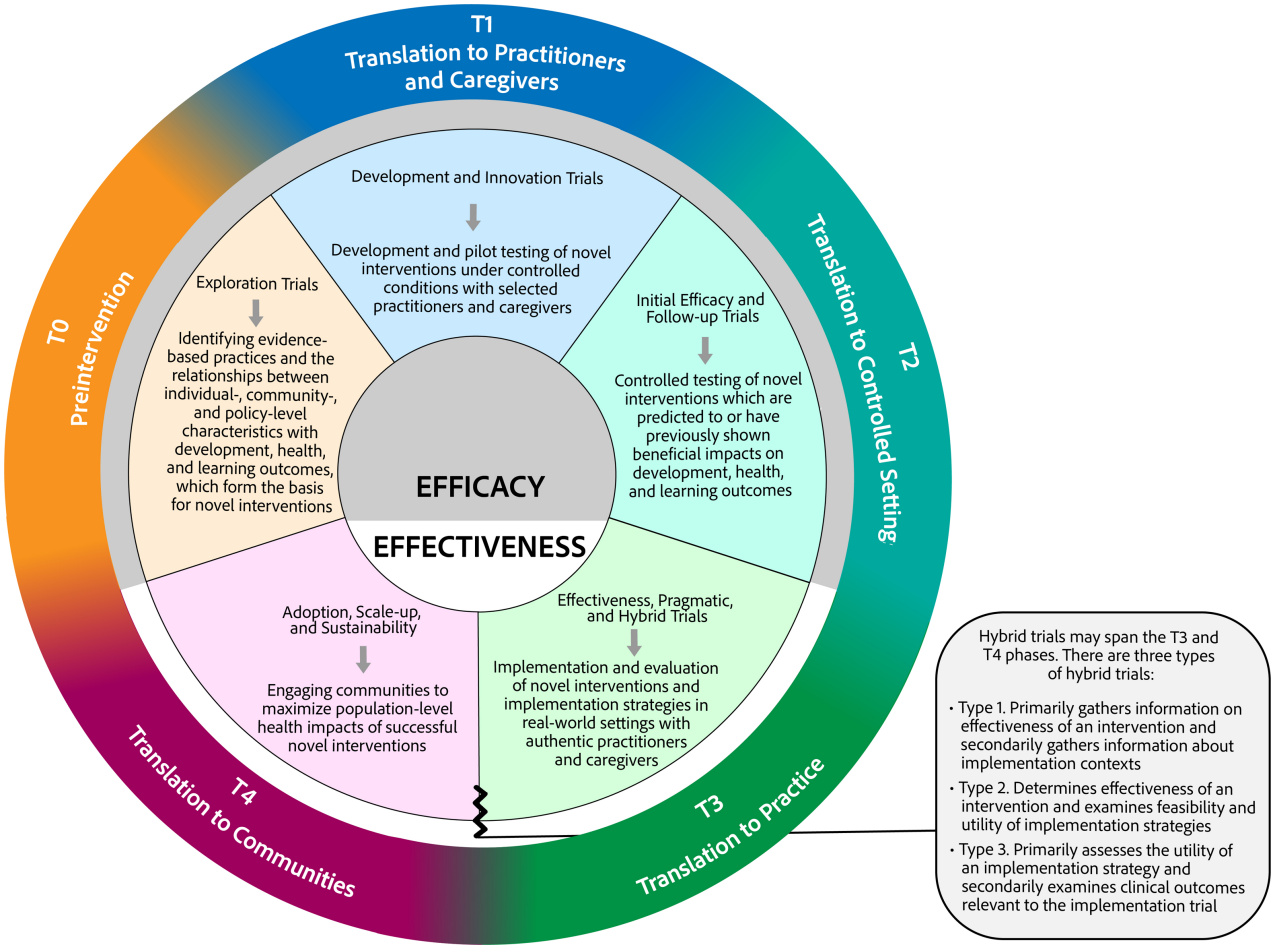
Proposed application of T0–T4 translational phases to early childhood intervention.

In an effort to demonstrate succinctly the alignments described above and to clarify the movement of “active ingredients” of an intervention from discovery to dissemination and implementation, we propose a contemporary translational framework for EI (Figure 1). We recognize there is an abundance of frameworks and models in the field of dissemination and implementation research, but given a translational approach to the development, implementation, and evaluation of interventions in EI has not yet been described, it is useful to propose a model tailored for EI. Our model is adapted from the UAMS framework (4) and informed by the work of Trivette and Dunst (21) and the IES trial descriptions (23–25). Modifications to the alignment offered by Trivette and Dunst (21) provide an updated depiction of the movement of EBPs from T0 to T4. The most significant change is the addition of a T0 phase (“Preintervention”) in which sources of evidence are gathered, analyzed, and evaluated to form a basis for novel interventions. As in the UAMS model, the phases are not standalone and rely on each of the other phases to provide information or data to support progression into the next phase or reevaluation and return to a previous phase. Each phase includes the relevant IES trial type, in parallel with the inclusion of NIH trial types in the UAMS model. Brief descriptions of key activities are included in each phase to clarify the types of work that occur throughout the continuum.

### 3.1. Case examples

This section provides three case examples spanning the T2–T4 phases to clarify the organization of EI research across later phase translational trials and demonstrate the distinction between efficacy and effectiveness.

T2. An ongoing efficacy trial funded by IES (PI: Kaiser; R324A190177) examines the *EMT en Español* program, which is an adaptation of Enhanced Milieu Teaching (EMT) (30). At the time this commentary was developed, this trial was one of just 36 efficacy trials funded by IES that focuses on early intervention and early learning. This randomized controlled trial aims to understand the impacts of *EMT en Español* on children’s expressive and receptive language, in addition to caregiver use of naturalistic teaching strategies. The trial is implemented in conjunction with providers and families who participate in the Part C early intervention program in Tennessee, with intervention delivery occurring in homes and community settings. This is a T2 study given its focus on examining efficacy of an evidence-based program (i.e., EMT). EMT has demonstrated efficacy with English-speaking toddlers in previous IES-funded trials (PI: Kaiser, R324A090181; PI: Kaiser, R324A150094) and pilot data suggest this Spanish adaptation has positive effects on both child and caregiver communication outcomes. While the trial occurs in real-world contexts, the provision of implementation supports beyond those offered in everyday practice and the controlled conditions under which the study is being conducted, consistent with IES standards, makes it an efficacy trial.

T3. Luoto et al. (31) conducted an implementation evaluation of *Msingi Bora (“Good Foundation”)*, a parenting intervention developed to support positive parenting practices and child development outcomes for children birth to age 2 and their families (Clinical Trial NCT03548558). This mixed methods study aimed to understand intervention inputs (e.g., program resources placed ahead of implementation, program content), outputs (e.g., implementation fidelity, parent attendance), and outcomes (e.g., parent knowledge of child development, child social–emotional development). Community Health Volunteers (CHVs) in participating villages in rural Kenya, who were members of their communities, served as the intervention delivery agents. This is a T3 study given its focus on understanding the facilitators of successful implementation of an evidence-based program in real-world contexts (i.e., local CHVs delivering the intervention to children and families in their villages) to achieve positive outcomes for children and families.

T4. A qualitative study funded by the National Institute of Child Health and Human Development (NICHD) examined implementation factors which supported sustainment of the *Infant Feeding, Activity, and Nutrition Trial (INFANT)* program (32). *INFANT* was developed in Victoria, Australia and focuses on addressing risk behaviors related to obesity for children aged 3–18 months and their families. *INFANT* was previously examined through a randomized controlled trial, then scaled to community-level implementation. This qualitative study aimed to clarify facilitators and barriers of *INFANT* sustainment according to the Consolidated Framework for Implementation Research (CFIR) and involved surveying past and current providers of *INFANT*. Findings will inform large scale implementation and support program adoption and continued sustainment. This is a T4 study given its focus on understanding factors of successful program scale-up across local contexts.

## 4. Discussion

The main goal of translational research is to produce meaningful results and clinically relevant outcomes which have a direct benefit to human health (4). At present, translational research is primarily considered in the fields of public health and medicine, without widespread application to research areas with strong behavioral foundations, like EI. However, EI researchers are increasingly engaged in translational research and implementation science. Even early efforts in the development of translational frameworks recognized that “the success of translational research is dependent upon the ability of researchers from different disciplines and backgrounds to pool their knowledge, skills, and resources and to work with communities in need to develop interventions that are amenable for use in diverse populations” (33, p. 6). The field of EI is inherently transdisciplinary, and young children and families are typically embedded within complex systems of supports and services which include professionals from the health, mental health, early learning, and family supports disciplines. These professionals strive to holistically support or enhance the unique strengths of each child and family in developmentally appropriate and culturally responsive ways. Translational research is necessary but not unique to the discovery of drugs or applications to medicine and health. We assert that to advance research and inform practice and policy in EI, it is more important than ever to build or strengthen connections and develop common ground for researchers across a variety of fields to understand where EI research intersects. The alignments described in Table 1 serve as a starting point for future transdisciplinary connections and conversations. Translational research cannot be done alone, by any singular discipline. Nevertheless, it can and should have novel applications to capture the movement of unique “active ingredients” of the diverse set of interventions relevant to EI. Enhancing the ways we engage in transdisciplinary research to reflect a translational and implementation focus will amplify the benefit of EI research. Most importantly, this shift will speed the movement of scientific evidence into the hands of the practitioners, families, and communities who will ultimately benefit from it.

## Data availability statement

The original contributions presented in the study are included in the article/Supplementary material, further inquiries can be directed to the corresponding author.

## Author contributions

KS, RS, and PS contributed to conception and design of this commentary. KS wrote and developed the manuscript and proposed framework with input from all authors. All authors contributed to the article and approved the submitted version.
